# A New Exhaustive Method and Strategy for Finding Motifs in ChIP-Enriched Regions

**DOI:** 10.1371/journal.pone.0086044

**Published:** 2014-01-24

**Authors:** Caiyan Jia, Matthew B. Carson, Yang Wang, Youfang Lin, Hui Lu

**Affiliations:** 1 School of Computer and Information Technology & Beijing Key Lab of Traffic Data Analysis, Beijing Jiaotong University, Beijing, China; 2 Department of Bioengineering/Bioinformatics, University of Illinois at Chicago, Chicago, Illinois, United States of America; 3 Center for Healthcare Studies, Institute for Public Health and Medicine, Northwestern University Feinberg School of Medicine, Chicago, Illinois, United States of America; 4 Division of Health and Biomedical Informatics, Department of Preventive Medicine, Northwestern University Feinberg School of Medicine, Chicago, Illinois, United States of America; 5 Shanghai Institute of Medical Genetics, Shanghai Children’s Hospital, Shanghai JiaoTong University, Shanghai, China; University of Georgia, United States of America

## Abstract

ChIP-seq, which combines chromatin immunoprecipitation (ChIP) with next-generation parallel sequencing, allows for the genome-wide identification of protein-DNA interactions. This technology poses new challenges for the development of novel motif-finding algorithms and methods for determining exact protein-DNA binding sites from ChIP-enriched sequencing data. State-of-the-art heuristic, exhaustive search algorithms have limited application for the identification of short (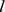, 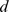) motifs (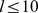, 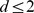) contained in ChIP-enriched regions. In this work we have developed a more powerful exhaustive method (FMotif) for finding long (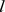, 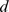) motifs in DNA sequences. In conjunction with our method, we have adopted a simple ChIP-enriched sampling strategy for finding these motifs in large-scale ChIP-enriched regions. Empirical studies on synthetic samples and applications using several ChIP data sets including 16 TF (transcription factor) ChIP-seq data sets and five TF ChIP-exo data sets have demonstrated that our proposed method is capable of finding these motifs with high efficiency and accuracy. The source code for FMotif is available at http://211.71.76.45/FMotif/.

## Introduction

Protein-DNA interactions play key roles in several cellular processes and functions including DNA transcription, packaging, replication, and repair. Identification of regions such as transcription factor binding sites (TFBSs), which are targeted by proteins called transcription factors (TFs), is crucial for a better understanding of transcriptional regulation. Although traditional footprinting assays can accurately identify the precise binding sites of any factor, this low-throughput method is highly technical and can only be used to analyze a single small region (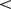
1 kilobase pairs (kb)) at a time. Chromatin immunoprecipitation followed by high-throughput deep sequencing (ChIP-seq) enables genome-wide detection of transcription factor binding sites as well as the localization of epigenetic regulatory markers on a genomic scale [1], [2]. It typically returns millions of short (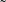
35–50 base pairs (bps)) sequence tags mapped onto a reference genome from a sample organism. Putative binding sites with high confidence can be extracted from peak-enriched regions in the genome by peak-calling programs [3]. However, the resolution of binding regions identified from ChIP-seq can be a few hundred base pairs and is one or two orders of magnitude larger than a typical TFBS. By using an exonuclease that trims DNA regions at a precise distance from binding sites, the novel ChIP-seq technique ChIP-exo is able to locate binding sites at high resolution [4]. However, according to the results in Rhee and Pugh [4], binding regions identified from ChIP-exo experiments may be tens of bps away from the exact binding locations, although some of them at the location indicated by the experiments. Computational methods are still needed to identify the exact binding locations of a TF in ChIP-seq or ChIP-exo data sets.

Binding sites for a specific TF are often highly conserved and have strong evidence for sequence specificity [5]. An actual DNA region interacting with and bound by a single TF usually ranges in size from 8–10 to 16–20 bps. In the past two decades, numerous programs have been developed to identify over-represented DNA sequence motifs from the promoters of co-regulated or homologous genes [6]. These programs can be divided into two groups. The first includes profile-based methods such as CONSENSUS [7], MEME [8], Gibsampler [9], AlignACE [10], PROJECTION [11], and CRMD [12], each of which attempts to maximize a statistic- or entropy-related score from a profile matrix (also called a position weight matrix (PWM)). The second group is comprised of consensus-based methods, which include SPELLER [13], WEEDER [14], [15], MITRA-count [16], Voting [17], PMSprune [18], WINNOWER [19], iTriplet [20], VINE [21], Stemming [22], and RecMotif [23]. These progams are designed to find potential 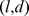 motifs within DNA sequences [19], where 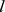 is the length of a motif and 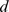 is the maximum number of mutations between a predicted binding site and the motif consensus. In most cases, profile-based methods are faster but suffer from lower accuracy due to their tendency to be trapped in a local optimum. Consensus-based methods are more accurate but slower due to the exponential growth of the search space with increasing values of 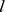 and 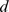.

Consensus-based methods can be further divided into two categories: pattern-driven and sample-driven approaches [16]. A pattern-driven approach attempts to enumerate all possible 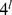 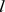-mer motifs with lexical order, while a sample-driven approach tries to test all possible (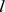, 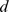) motifs generated from real 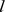-mers of input sequences. For the methods mentioned above, SPELLER, WEEDER, and MITRA-count are pattern-driven approaches and Voting, PMPprune, WINNOWER, iTriplet, VINE, Stemming, and RecMotif are sample-driven. By using pattern-driven approaches (with the exception of MITRA-count), one can automatically find planted (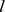, 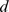) motifs without prior knowledge of the length 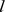. On the contrary, sample-driven approaches require that 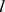 be specified for each run. In real applications, the exact length of motifs contained in a set of sequences is usually unknown. The pattern-driven algorithm WEEDER has been successful in real eukaryotic applications [24] but has not been improved upon to the best of our knowledge. In this study, we have developed a more powerful method to extract 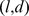 motifs and their binding locations contained in DNA sequences without prior knowledge of motif length and have used this method to identify motifs and their binding locations in ChIP-enriched regions.

The pattern-driven approach MITRA-count builds a mismatch tree for all 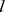-mers first, then traverses search space recursively from the root down in depth-first order. Therefore, the length 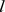 of a predicted motif must be specified in advance. SPELLER enumerates all possible motifs in a depth-first manner throughout the search space, then scans and counts all possible instances of the current motif with length 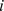 (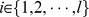) from the suffix tree of input sequences. The algorithm can identify planted (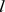, 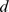) motifs efficiently when 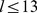 and 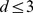 (see Table 1). In order to increase the speed of SPELLER, WEEDER includes an error ratio 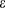 (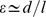) for the algorithm that narrows the search space such that for all 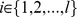 the number of mismatches between the first 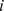 nucleotides of a candidate 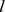-mer motif and the first 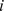 nucleotides of a valid instance of the motif is at most 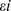. The algorithm can accelerate SPELLER to some extent, especially when 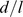 is small (e.g., 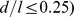. Unfortunately, not all motif occurrences satisfy this restriction. WEEDER must lower the occurrence frequency 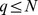 to make sure exact motifs will not be missed. However, WEEDER’s run time increases dramatically with the decrease of 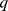. For instance, for (15, 4), 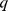 should be lowered to half the number of sequences at the OOPS constraint (one occurrence(s) of the motif instance(s) per sequence) to make sure that the true motif will be discovered [14]. However, WEEDER’s run time may be even longer than SPELLER under the condition that the two algorithms use the same programming techniques. Thus, a more efficient method is needed to improve the efficiency of pattern-driven algorithms without knowledge of the length of predicted motifs under the ZOMOPS constraint (zero, one or multiple occurrence(s) of the motif instance(s) per sequence).

**Table 1:** Comparisons between FMotif and other pattern-driven algorithms on (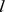, 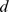) samples with 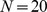, 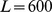, and 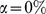 noise sequences.

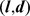 ; 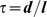	SPELLER	WEEDER( 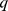 )	MITRA	FMotif
(10, 2); 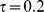	17.16 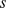 -1	7.47 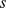 (19)-1	1.83 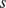 -2	0.59 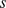 -1
(11, 2); 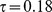	17.73 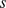 -1	32.53 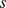 (15)-1	1.82 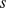 -1	0.59 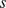 -1
(12, 3); 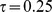	4.42 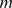 -1	9.35 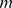 (15)-1	21.22 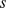 -1	6.77 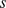 -1
(13, 3); 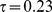	4.42 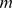 -1	2.80 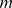 (18)-1	21.25 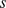 -3	6.73 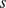 -1
(14, 4); 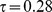	1.05 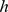 -1	2.41 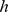 (15)-1	3.94 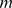 -1	1.32 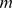 -1
(15, 4); 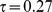	1.05 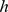 -1	1.08 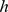 (16)-1	3.93 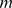 -1	1.31 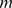 -1
(15, 5); 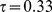	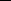	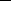	41.25 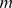 -2	15.51 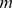 −/
(16, 5); 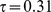	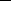	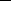	41.19 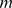 -1	15.50 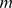 -1
(17, 6); 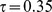	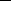	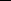	6.58 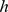 -1	3.17 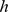 −/
(18, 6); 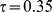	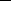	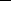	6.84 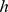 -1	3.17 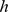 -1

‘WEEDER(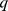)’ indicates the execution time of WEEDER given the occurrence frequency threshold 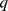. ‘
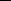
’ indicates a run time of over 10 hours. 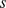, 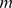, and 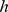 are the units of a run time and denote seconds, minutes, and hours respectively. The number after each run time is the ranking number of a true planted motif among the top 25 predicted motifs. ‘
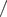
’ after a run time indicates that the real motifs were not in the top 25.

Additionally, the programs mentioned above are not computationally efficient enough to process a large number of ChIP-seq peaks. In recent years, several programs have been developed to cope with large-scale ChIP-seq data. Some are ChIP-tailored versions of previously-developed software (e.g., ChIP-MEME [25], DREME [26], and GimmeMotifs [27]). These typically restrict motif discovery to a few hundred peaks and usually ignore the remaining unselected sequences. Other programs are faster versions of previous software (e.g., STEME [28], ChIPMunk [29], and HMS [30]). STEME is a faster version of MEME and involves indexing sequences with a suffix tree, which accelerates the expectation-maximization (EM) steps. ChIPMunk combines EM with a greedy approach similar to CONSENSUS and decreases the run time of the optimization procedure. HMS is an improved version of Gibbs Sampler and combines stochastic sampling with deterministic, greedy search steps. Another group of programs integrate other information such as TFBS positional priors [31] or transcription start sites [32] in order to optimize a PWM of ChIP-enriched regions. As mentioned above, these programs still have a local optimum problem. Similar to SPELLER and WEEDER, some of these programs are consensus-based methods (sometimes called word enumeration methods). These include RSAT [33], Cisfinder [34] and POSMO [35]. RSAT is a word enumeration method and has been developed to process whole ChIP-seq peak data sets, but is limited to short (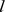, 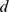) motifs (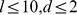). Cisfinder is a word clustering method and combines short 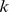-mer enumeration (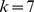, 8, or 9) with a clustering strategy. POSMO, also a word clustering method, uses TFBS positional bias information along with 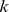-mer enumeration and clustering. However, both Cisfinder and POSMO use clustering methods to group short 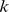-mers and therefore cannot find exact (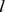, 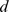) motifs contained in sequences. Thus, finding exact (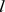, 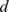) motifs with larger values of 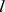 and 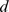 in a large-scale sequence data set is still very difficult.

According to a previous study by Keich and Pevzner [36], real signals may be mixed with spurious motifs contained in background sequences under the OOPS constraint when the degenerative ratio 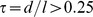. A larger 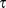 makes it more difficult to discriminate between a real motif and spurious motifs. However, some sequences may not contain any occurrence of a motif. As previously mentioned, we have concentrated on a more generalized model (the ZOMOPS constraint). Under this constraint, we have found that, except for the degenerative ratio 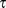, the ratio of noise sequences 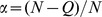, where 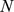 is the number of sequences and 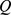 is the number of sequences containing at least one variant of a motif, negatively affects (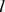, 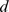) motif searches. A larger 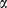 leads to more spurious motifs in background sequences. It is suspected that 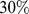 of factor-bound locations in ChIP-seq data may be false positives [4]. Plus, there may be different versions of DNA-binding motifs for any given TF. A specified motif may only occur in 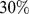 of binding regions. Although false positive rates in ChIP-seq data sets are low enough that statistical conclusions can be drawn in most cases, the noise (plus the diversity of DNA-binding modes) still interrupts the motif-finding process and alters motif-finding results. Thus, this may not be the best way to identify motifs in full-size ChIP-seq data sets. After running a peak-calling program on a raw ChIP-seq data set, peaks along with their ChIP enrichment values, 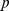-values, or false discovery rates (FDRs) can be obtained. False positive peaks are those with low peak enrichment values, 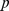-values, or FDRs. A better method may be to find motifs with a high confidence value (i.e., those that are plentiful enough to draw statistical conclusions) in peak-enriched regions and subsequently scan their binding locations with the degenerative value 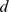 in the remaining peak regions that have low peak enrichment values, 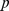-values, or FDRs. This would not only exclude more noise and spurious motifs [37], but it would also take advantage of well-developed motif-finding tools with an acceptable level of scalability. A similar idea was used in MICSA and achieved good performance [38]. However, MICSA used the optimal method MEME (the accuracy of which is limited [12], [19]) to get the PWM of a motif for only the first three hundred peak-enriched regions.

In this study we have found that for motifs with length 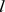, both SPELLER and WEEDER have been designed to check each 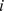-mer (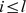) in the pattern space with depth-first order and count the variants of the 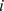-mer in the suffix tree of sequences from the root to layer 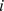. The suffix tree is scanned one time for each 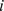-mer pattern. Thus, as 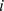 increases, the algorithms scan the suffix tree an increasing number of times. In fact, the mismatch information in layer 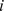 of a suffix tree can be used to search for (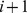)-mers in the pattern space. For this reason, we constructed a new suffix tree structure with mismatch information (called a mismatched suffix tree) and developed a fast motif enumerative method (FMotif) under the ZOMOPS constraint. Using the newly constructed suffix trees, we incorporated the mismatch information in layer 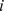 of the mismatched suffix trees to verify (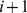)-mers in the pattern space. We then updated mismatch information in layer 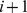 of the mismatched suffix trees. In this way we were able to implement a depth-first search within the pattern space and the mismatched suffix trees simultaneously. To process large-scale ChIP-seq data sets, we integrated the peak detection method MACS [39] with our motif-finding method and ChIP-enriched sampling strategy, which allowed us to locate the exact binding locations in ChIP-seq and ChIP-exo data sets. We chose MACS because it has been shown to perform well when compared to several other peak-calling programs [3].

## Results

### Experimental Results on Artificial Data Sets

We compared FMotif with the existing pattern-driving methods including SPELLER, WEEDER, and MITRA-count (MITRA for short) on synthetic samples to show the efficiency of our proposed method. All synthetic samples were generated following the method of Pevsner and Sze [19], where 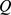 (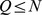) variants of an 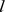-length motif were randomly planted into 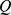 sequences selected randomly from a set of 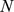 sequences with length 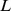. In this (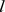, 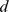) model, each planted variant of the motif with length 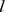 had exactly 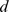 mismatches with the motif itself.

In the first group of experiments, we tested the performance of these algorithms on (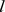, 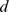) sample sets without noise sequences (i.e., 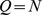) at standard settings, where the number 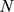 and the length 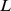 of sequences are set to 20 and 600, respectively [14], [16], [19]. These test results are shown in Table 1. ‘WEEDER(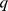)’ indicates the execution time of WEEDER given the occurrence frequency threshold 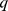. ‘
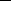
’ indicates a run time of over 10 hours. 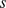, 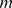, and 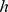 denote seconds, minutes, and hours respectively. The number after each run time is the ranking number of a true planted motif among the top 25 predicted motifs. ‘
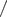
’ after a run time indicates that the real motifs were not in the top 25. In the second group of experiments, we first tested the influence of the ratio of noise sequences 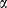 (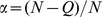) on (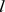, 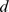) samples using FMotif with typical settings (i.e., 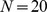 and 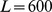). In order to provide a more comprehensive comparison of the calculation speed when noise was added, we compared FMotif to SPELLER and MITRA on (10, 2), (11, 2), (12, 3), and (13, 3) samples. We avoided comparisons over motifs more complicated than (13, 3) because SPELLER lacked computational efficiency on these problems and WEEDER required tuning of parameter 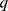. These test results are shown in Table 2, where 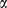 is set at 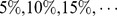, and 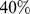, ‘
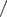
’ indicates that the real motifs were not in the top 25, and the first line for (10, 2), (11, 2), (12, 3) and (13, 3) is the FMotif result, the second line (denoted by ‘M..’) is the MITRA result, and the third line (denoted ‘S..’) is the SPELLER result. We then tested the influence of the noise ratio 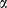 on samples with 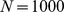 and 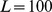 to simulate ChIP-enriched regions because those regions are usually relatively short and the number of regions is usually large. We subsequently compared FMotif to SPELLER and MITRA on (10, 2), (11, 2), (12, 3), and (13, 3) samples as before. These test results are shown in Table 3, where 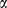 is set at 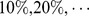, and 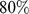. In the third group of experiments, we tested FMotif scalability using two groups of samples to see whether it was suitable for recognizing motifs in large-scale ChIP-enriched regions. The settings of the first group were 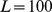, 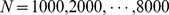 and no noise sequences (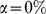). These test results are shown in Table 4. The settings of the second group were 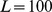, 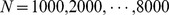 and 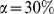 noise sequences in order to mimick ChIP-seq data. These test results are shown in Table 5. All experiments were performed on a computer with an Intel 2.99 GHz processor, 2.00GB of main memory, and the Windows XP operating system.

**Table 2 pone-0086044-t002:** Results for noise-influenced models on (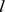, 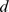) samples with 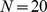, 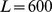, and 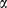 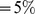, 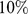, 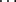, 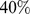 noise sequences.

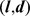	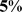	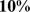	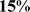	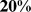	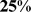	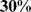	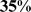	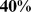
(10, 2)	0.78 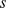 -1	0.95 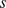 -1	1.06 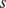 -1	1.19 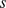 -1	1.30 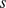 -1	1.44 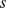 -3	1.63 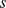 -5	/
M..	2.78 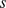 -1	3.05 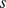 -1	3.23 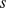 -1	3.44 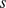 -1	3.78 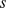 -1	4.83 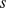 -3	5.23 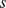 -15	/
S..	38.99 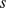 -1	1.09 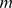 -1	1.53 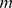 -1	1.96 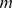 -1	2.51 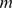 -1	3.34 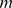 -3	4.69 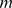 -21	/
(11, 2)	0.77 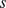 -1	0.94 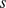 -1	1.06 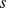 -1	1.17 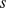 -1	1.30 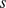 -1	1.44 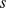 -1	1.61 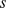 -1	1.83 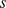 -1
M..	2.77 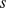 -1	3.05 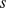 -1	3.23 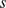 -1	3.23 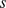 -1	3.44 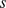 -1	3.77 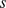 -1	4.38 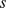 -1	5.19 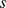 -1
S..	38.13 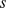 -1	1.08 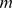	1.52 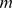 -1	1.95 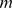 -1	2.48 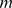 -1	3.31 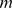 -1	4.66 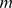 -1	6.53 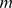 -1
(12, 3)	9.16 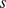 -1	11.69 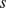 -1	13.92 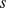 -1	15.88 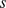 -1	17.61 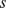 -6	/	/	/
M..	33.13 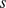 -1	33.23 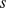 -1	39.28 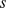 -1	43.45 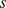 -1	46.64 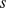 -4	50.36 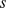 -20	/	/
S..	9.78 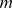 -1	17.69 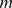 -1	26.94 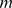 -1	36.16 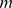 -1	45.64 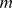 -2	58.11 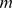 -8	/	/
(13, 3)	9.17 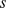 -1	11.64 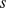 -1	13.95 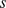 -1	15.88 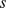 -1	17.63 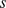 -1	19.50 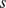 -5	21.83 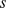 -2	24.80 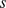 -1
M..	27.23 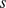 -1	34.38 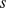 -1	40.22 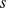 -1	43.44 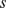 -1	46.66 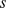 -1	50.58 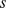 -1	57.14 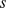 -1	1.12 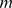 -1
S..	9.80 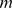 -1	17.71 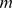 -1	27.07 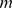 -1	36.12 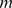 -1	45.80 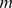 -1	58.33 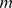 -1	1.30 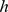 -1	1.79 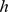 -1
(14, 4)	1.85 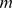 -1	2.33 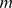 -1	2.89 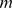 -9	3.47 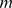 -5	/	4.52 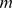 -20	/	/
(15, 4)	1.82 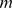 -1	2.32 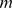 -1	2.89 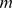 -1	3.48 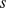 -1	4.03 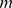 -1	4.52 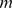 -3	5.02 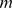 -3	5.66 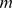 -1
(15, 5)	/	/	/	/	/	/	/	/
(16, 5)	22.90 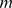	29.52 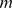 -1	36.05 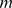 -1	/	/	/	/	/
(17, 6)	/	/	/	/	/	/	/	/
(18, 6)	5.18 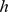	7.07 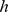 -1	/	/	/	/	/	/

The ratio of noise sequences 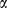 is set at 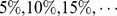, and 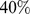, ‘
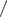
’ indicates that the real motifs were not in the top 25. The number after each run time is the ranking number of a true planted motif among the top 25 predicted motifs. The first line for (10, 2), (11, 2), (12, 3) and (13, 3) is the FMotif result, the second line (denoted by ‘M..’) is the MITRA result, and the third line (denoted ‘S..’) is the SPELLER result. 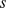, 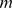, and 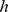 denote seconds, minutes, and hours respectively.

**Table 3 pone-0086044-t003:** Results for noise-influenced models on (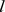, 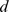) samples with 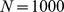, 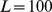, and 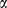 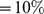, 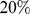, 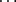, 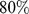 noise sequences.

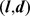	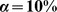	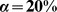	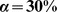	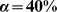	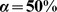	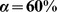	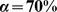	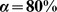
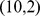	6.39 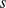 -1	7.63 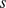 -1	10.36 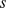 -1	12.95 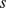 -1	13.63 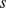 -1	14.64 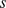 -1	23.13 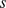 -1	24.84 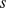 -6
M..	12.44 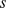 -1	12.49 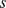 -1	19.38 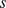 -1	31.61 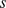 -1	31.97 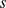 -1	32.29 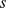 -1	1.47 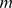 -1	1.64 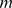 -1
S..	1.23 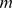 -1	2.29 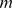 -1	6.84 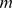 -1	10.68 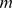 -1	12.72 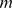 -1	15.03 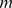 -1	1.05 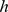 -1	1.23 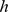 -1
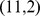	6.41 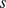 -1	7.70 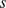 -1	10.28 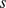 -1	12.59 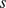 -1	13.60 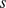 -1	14.66 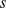 -1	23.01 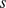 -1	24.86 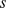 -2
M..	12.41 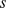 -1	12.66 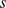 -1	18.58 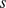 -1	31.44 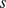 -1	31.86 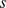 -1	32.16 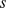 -1	1.41 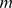 -1	1.64 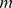 -1
S..	1.03 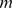 -1	2.09 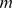 -1	5.86 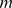 -1	10.64 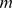 -1	12.74 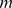 -1	15.08 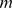 -1	1.05 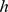 -1	1.23 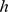 -1
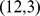	1.19 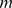 -1	1.44 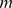 -1	1.66 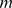 -1	2.40 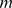 -1	2.94 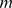 -1	3.25 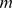 -1	3.56 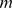 -1	/
M..	2.44 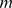 -1	2.84 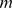 -1	2.87 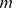 -1	4.55 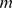 -1	7.92 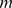 -1	7.98 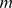 -1	8.63 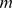 -1	25.91 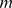 -1
S..	15.45 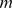 -1	32.14 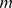 -1	45.25 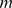 -1	2.22 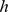 -1	3.80 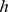 -1	4.42 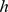 -1	6.00 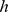 -1	22.34 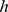 -1
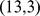	1.18 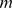 -1	1.43 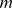 -1	1.66 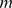 -1	2.33 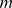 -1	2.91 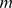 -1	3.50 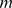 -1	3.56 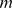 -1	5.72 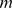 -10
M..	2.33 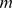 -1	2.84 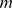 -1	2.87 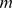 -1	4.31 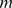 -1	7.90 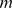 -1	7.96 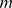 -1	8.41 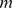 -1	25.90 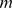 -1
S..	15.39 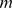 -1	32.30 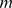 -1	46.20 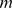 -1	2.20 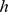 -1	3.82 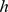 -1	4.40 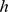 -1	5.92 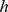 -1	22.25 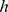 -1
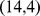	10.03 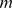 -1	16.35 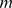 -1	19.17 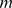 -1	21.63 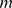 -1	30.65 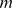 -1	42.05 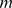 -1	45.05 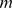 -1	/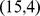	10.07 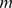 -1	16.36 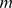 -1	19.19 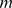 -1	21.78 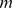 -1	30.67 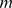 -1	42.02 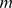 -1	45.04 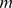 -1	/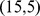	1.69 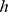 -1	2.23 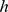 -1	3.84 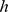 -1	4.66 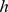 -1	5.18 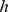 -1	7.38 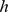 -1	10.60 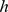 -13	/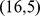	1.70 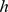 -1	2.24 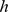 -1	3.89 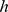 -1	4.67 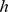 -1	5.20 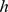 -1	7.38 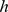 -1	10.61 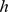 -3	/

The ratio of noise sequences 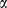 is set at 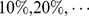, and 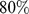. Row definitions are the same as those in Table 2.

**Table 4 pone-0086044-t004:** A demonstration of FMotif scalability on (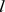, 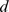) samples for 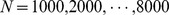 sequences, 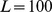, and no (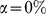) noise sequences.

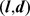	1000	2000	3000	4000	5000	6000	7000	8000
(10, 2)	3.34 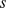 -1	8.05 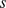 -1	11.90 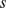 -1	15.80 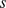 -1	19.58 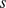 -1	23.34 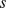 -1	26.83 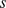 -1	42.81 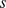 -1
(11, 2)	3.36 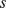 -1	8.08 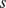 -1	11.86 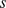 -1	15.61 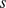 -1	19.45 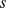 -1	23.16 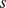 -1	26.84 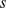 -1	39.25 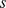 -1
(12, 3)	33.41 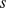 -1	1.31 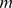 -1	1.84 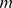 -1	2.38 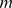 -1	2.78 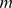 -1	3.32 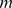 -1	4.03 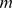 -1	4.46 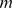 -1
(13, 3)	34.62 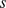 -1	1.30 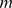 -1	1.85 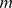 -1	2.40 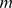 -1	2.84 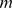 -1	3.32 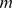 -1	3.82 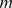 -1	4.33 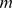 -1
(14, 4)	4.51 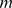 -1	10.22 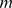 -1	15.14 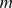 -1	19.93 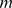 -1	24.85 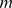 -1	29.54 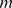 -1	34.25 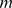 -1	39.03 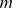 -1
(15, 4)	4.52 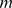 -1	10.30 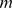 -1	15.23 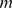 -1	20.05 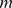 -1	24.87 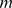 -1	29.16 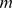 -1	34.38 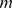 -1	39.26 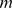 -1
(15, 5)	35.06 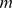 -1	1.46 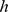 -1	2.15 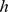 -1	2.68 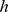 -1	3.21 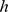 -1	3.75 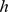 -1	4.27 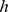 -1	4.77 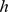 -1
(16, 5)	35.02 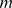 -1	1.47 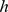 -1	2.13 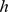 -1	2.70 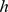 -1	3.24 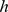 -1	3.84 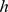 -1	4.27 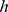 -1	4.75 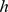 -1

The number after each run time is the ranking number of a true planted motif among the top 25 predicted motifs. 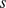, 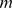, and 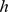 denote seconds, minutes, and hours respectively.

**Table 5:** A demonstration of FMotif scalability on (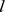, 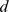) samples for 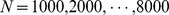 sequences, 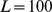, and 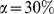 noise sequences.

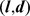	1000	2000	3000	4000	5000	6000	7000	8000
(10, 2)	10.33 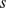 -1	26.19 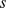 -1	45.16 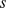 -1	1.10 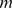 -1	1.63 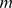 -1	2.09 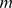 -1	2.70 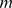 -1	2.97 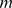 -1
(11, 2)	10.33 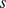 -1	25.89 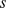 -1	45.30 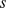 -1	1.09 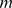 -1	1.49 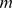 -1	1.90 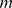 -1	2.43 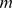 -1	2.88 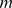 -1
(12, 3)	1.66 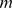 -1	4.13 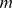 -1	7.26 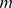 -1	10.70 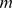 -1	14.07 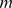 -1	17.52 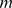 -1	21.40 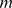 -1	25.76 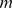 -1
(13, 3)	1.66 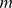 -1	4.15 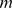 -1	7.27 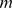 -1	10.72 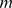 -1	14.06 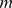 -1	17.56 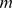 -1	21.51 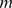 -1	25.94 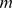 -1
(14, 4)	19.14 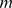 -1	45.85 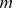 -1	1.28 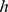 -1	1.90 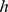 -1	2.59 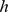 -1	3.24 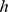 -1	3.88 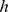 -1	4.52 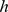 -1
(15, 4)	19.16 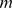 -1	45.81 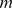 -1	1.29 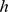 -1	1.91 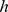 -1	2.59 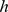 -1	3.25 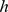 -1	3.89 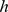 -1	4.54 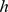 -1
(15, 5)	3.86 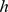 -1	8.83 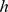 -1	14.70 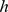 -1	21.28 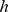 -1	28.40 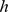 -1	35.48 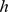 -1	43.08 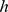 -1	50.36 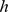 -1
(16, 5)	3.87 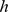 -1	8.61 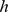 -1	14.64 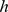 -1	20.81 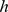 -1	28.52 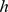 -1	35.15 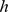 -1	43.35 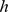 -1	51.22 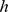 -1

Row and column definitions are the same as those in Table 4.

The results in Tables 1–5 lead to three observations. First, FMotif is a fast and exact algorithm and capable of finding (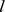, 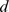) motifs in synthetic samples without being given the length 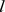 of a predicted motif. It performs faster than SPELLER, MITRA, and WEEDER without sacrificing accuracy. As mentioned above, WEEDER’s efficiency suffers significantly (see (14, 4) in Table 1 for an example) when the occurrence frequency threshold 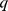 is too low, and MITRA requires that the length 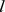 be specified a priori. It should be noted that FMotif ranked all motifs with different lengths together by significance score. For the samples whose true motifs were not ranked in the top 25, the top motifs were usually 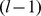
- or 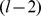
- substrings of the true motifs with length 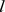. In these cases the true motifs were still in the output list but were ranked below the top 25. Second, noise sequences have a strong effect on the results and the speed of the method. With an increase in 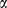, the run time increases as well. Like the degenerative ratio 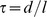, the ratio of noise sequences 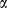 also weakens motifs, especially when background sequences are long (see Table 2). Spurious motifs in background sequences bury the authentic signals when either 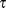 or 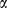 is large. For example, real motifs were difficult to filter by their significance score for the (15, 5) motif in Table 2, even when the noise sequence ratio was set to 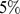. In this case, many spurious motifs of length 14–15 with a large significance score were ranked among the top 25. When the length of background sequences was shorter and the number of sequences was larger, the signals were stronger and could be easily identified even if a large portion of noise sequences was added (see Table 3). This is consistent with the previous result that false-positives could be reduced by decreasing the sequence length or by adding more sequences to the data set [37]. Third, as shown in Tables 4 and 5, FMotif is capable of operating on a large scale even when there are 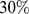 noise sequences in samples. This allowed us to use FMotif to process peak regions within ChIP-seq and ChIP-exo data sets.

Additionally, we compared FMotif with CisFinder, which uses 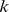-mer enumeration with 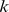-mer clustering to find motifs in large-scale ChIP-seq peak regions. Using both algorithms, we verified the accuracy of FMotif and CisFinder by searching for long (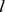, 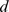) motifs in synthetic sample sets with 3000 sequences, each of which contained a planted variant of a parent motif. The experimental results are shown in Table 6, where ‘Planted Motif’ indicates a planted motif consensus in a set of sequences, ‘FMotif (*Top-1*)’ indicates the top ranked motif consensus found by FMotif in a sample set, ‘CisFinder’ indicates the closest matching motif consensus (described by IUPAC nucleotide codes) found by CisFinder in a sample set, ‘
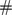
’ indicates the number of variants of a reported motif found by FMotif or CisFinder in a sample set, and ‘Rank’ after ‘
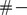
’ is the ranking number of the reported motif found by Cisfinder in Table 6.

**Table 6:** Comparisons between FMotif and CisFinder on large (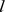, 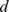) samples with 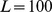, 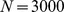, and no (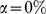) noise sequences.

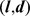	Planted Motif	FMotif (*Top-1*)	CisFinder ( 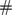 -Rank)
		( 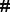 )–( 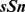 )–( 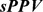 )	( 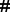 -Rank)–( 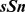 )–( 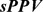 )
(10, 2)	CACGAGAACC	CACGAGAACC	CACGANAACC
		(3108)–(1.0)–(0.97)	(66-1)–(0.02)–(0.98)
(11, 2)	TTGACAAGGAT	TTGACAAGGAT	TTVACAASGA
		(3026)–(1.0)–(0.99)	(186-1)–(0.06)–(0.96)
(12, 3)	TCCATTAGGTGG	TCCATTAGGTGG	CCWMCTAABKGAMC
		(3089)–(1.0)–(0.97)	(80-1)–(0.02)–(0.93)
(13, 3)	CGATAGGTCTATG	CGATAGGTCTATG	ATAGKYCTA
		(3026)–(1.0)–(0.99)	(148-1)–(0.05)–(0.96)
(14, 4)	AGCTATCTATTTAA	AGCTATCTATTTAA	TAAANWGATA
		(3161)–(1.0)–(0.95)	(75-1)–(0.02)–(0.85)
(15, 4)	GATCACACGGAAACC	GATCACACGGAAACC	CACACGGAAAC
		(3022)–(1.0)–(0.99)	(109-3)–(0.04)–(0.98)
(15, 5)	GGTGGGGCGGGCGAT	GGTGGGGCGGGCGAT	CMGGYYGGGKCG
		(3371)–(1.0)–(0.89)	(40-1)–(0.01)–(0.77)
(16, 5)	GAGGCTTGTAAACGTT	GAGGCTTGTAAACGTT	GGMGKGTAAAMGTTKC
		(3062)–(1.0)–(0.98)	(59-1)–(0.02)–(0.85)

‘Planted Motif’ indicates a planted motif consensus in a set of sequences, ‘FMotif (*Top-1*)’ indicates the top ranked motif consensus found by FMotif in a sample set, ‘CisFinder’ indicates the closest matching motif consensus (described by IUPAC nucleotide codes) found by CisFinder in a sample set, ‘
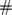
’ indicates the number of variants of a reported motif found by FMotif or CisFinder in a sample set, and ‘Rank’ after ‘
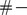
’ is the ranking number of the reported motif found by Cisfinder. The *site-level sensitivity* (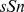) and *positive predictive value* (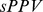) metrics described by Tompa [24] were used to statistically quantify the accuracy of the two methods, where 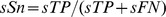 and 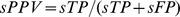, 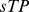 is the number of known sites overlapping predicted sites, 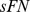 is the number of known sites not overlapping predicted sites, and 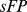 is the number of predicted sites not overlapping known sites. A predicted site overlaps a known site if they share at least a half of the length of known sites.

We used the *site-level sensitivity* (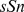) and *positive predictive value* (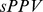) metrics described by Tompa [24] to statistically quantify the accuracy of the two methods, where 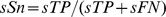 and 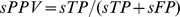, 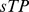 is the number of known sites overlapping predicted sites, 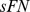 is the number of known sites not overlapping predicted sites, and 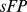 is the number of predicted sites not overlapping known sites. A predicted site overlaps a known site if they share at least a half of the length of known sites. In order to give a more comprehensive comparison of the accuracy of the two methods on simulated ChIP-seq data sets, we added 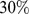 noise sequences to samples with 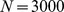 and 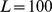 and performed the experiments again. These test results are shown in Table 7.

**Table 7:** Comparisons between FMotif and CisFinder on large (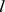, 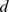) samples with 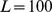, 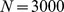, and 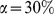 noise sequences.

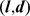	Planted Motif	FMotif (*Top-1*)	CisFinder
		( 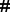 )–( 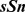 )–( 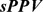 )	( 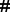 -Rank)–( 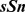 )–( 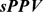 )
(10, 2)	TGACCCCACG	TGACCCCACG	YHGAYCHMACGSM
		(2192)–(1.0)–(0.96)	(65-2)–(0.03)–(0.89)
(11, 2)	GCGGTGTACCA	GCGGTGTACCA	GCGGTNTACC
		(2130)–(1.0)–(0.99)	(120-2)–(0.06)–(0.99)
(12, 3)	CACGGGCCTTAG	CACGGGCCTTAG	CAKSGGCCBBAG
		(2182)–(1.0)–(0.96)	(61-2)–(0.03)–(0.85)
(13, 3)	TTCAGTAAGCACG	TTCAGTAAGCACG	TTCRGTAARCAYG
		(2124)–(1.0)–(0.99)	(99-1)–(0.05)–(0.96)
(14, 4)	GCAAGTCACCGTGT	GCAAGTCACCGTGT	RVAAGTVVBNGTGT
		(2167)–(1.0)–(0.97)	(42-2)–(0.02)–(0.90)
(15, 4)	AAGGTGTTGGTATGG	AAGGTGTTGGTATGG	AARGTGTTGGTATGGG
		(2137)–(1.0)–(0.98)	(70-2)–(0.03)–(0.90)
(15, 5)	AATACTGTGCATGGA	AATACTGTGCATGGA	AATWCTGTSCA
		(2272)–(1.0)–(0.92)	(27-1)–(0.01)–(0.70)
(16, 5)	AGCTTGCCAGCGACGT	AGCTTGCCAGCGACGT	VGCTSKCCAGCWACGT
		(2145)–(1.0)–(0.98)	(51-1)–(0.02)–(0.90)

Column definitions are the same as those in Table 6.

As evident from Tables 6 and 7, FMotif is an exact algorithm. It reported all true motif consensuses and their planted variants plus false positive variants in background sequences. CisFinder performed quickly but suffered from low accuracy (due to low *sensitivity*), especially when 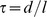 was large. FMotif and CisFinder both were robust after 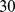 noise sequences were added to the samples. It should be pointed out that, although there are various resources on CisFinder’s website (http://lgsun.grc.nia.nih.gov/cis-finder/download.html), we used only the motif-finding program. There are other programs focused on motif clustering, motif improvement, motif comparison, and other tasks. If all programs were used together, a better motif and more of its binding sites may be identified. However, the CisFinder algorithm [34] was implemented in that motif-finding program and there was no direct way to use all these programs together based on our knowledge.

### Experimental Results Using ChIP-seq Data Sets

We tested FMotif using 12 mouse ChIP-seq data sets for 12 DNA-binding TFs (CTCF, cMyc, Esrrb, Klf4, Nanog, nMyc, Oct4, Smad1, Sox2, STAT3, Tcfcp2I1, and Zfx) involved in mouse embryonic stem cell pluripotency and self-renewal [40]. These ChIP-seq data sets have been deposited in the GEO database with ID number GSE11431. We also tested FMotif using four widely used human ChIP-seq data sets for four DNA-binding TFs including CTCF (CCCTC-binding factor [41], named CTCF(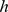) in the paper), FoxA1 (hepatocyte nuclear factor 3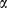 [42]), NRSF (neuron-restrictive silencer factor [2]), and STAT1 (signal transducer and activator of transcription protein [1]). The raw sequence of the FoxA1 ChIP-seq data set was downloaded from http://liulab.dfci.harvard.edu/MACS/Sample.html. The bed format of the CTCF, NRSF and STAT1 ChIP-seq data sets was downloaded from http://dir.nhlbi.nih.gov/papers/lmi/epigenomes/sissrs/. These downloaded short reads were mapped onto the newest version of mouse genome assembly mm10 and human genome assembly hg19, respectively. The peak regions were extracted from these reads using the peak finding program MACS [39] with a false discovery rate (FDR) threshold of 0.2. The reads were ranked by their FDR if a negative control was available, or by 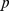-value otherwise. To prepare the data sets for use with motif discovery algorithms, we mapped the summits of the ChIP-seq peaks and extracted the 100 bps of genomic sequence centered around each peak.

**Figure 1:**
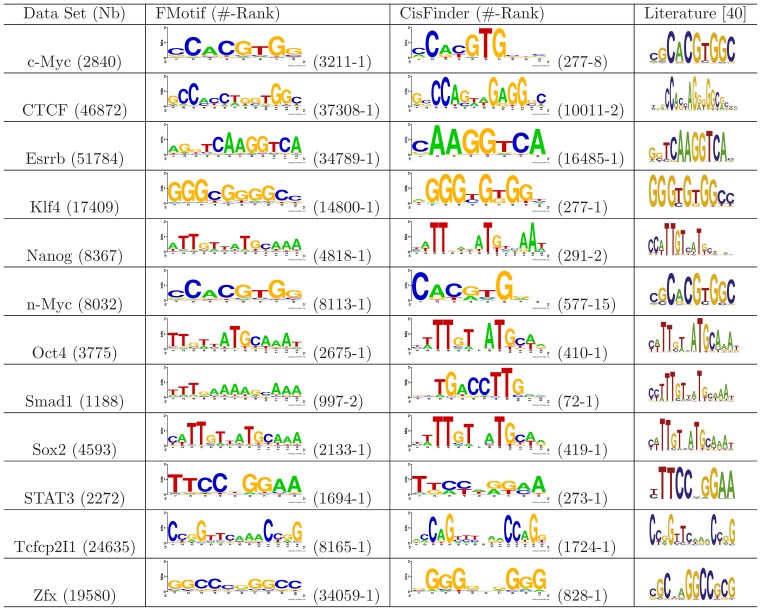
Motifs in 12 mouse ES Cell ChIP-seq data sets. FMotif was tested using mouse ChIP-seq data sets for 12 DNA-binding TFs (CTCF, cMyc, Esrrb, Klf4, Nanog, nMyc, Oct4, Smad1, Sox2, STAT3, Tcfcp2I1, and Zfx) involved in mouse embryonic stem cell pluripotency and self-renewal [40]. Results from CisFinder and published motifs in literature are shown for comparison. ‘Nb’ indicates the number of peak-enriched regions predicted by the peak-calling program MACS with an FDR threshold of 0.2 or a 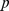-value threshold of 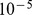, ‘FMotif’ and ‘CisFinder’ indicate the closest matching motif logos found by these programs (all motif logos were generated using the web-based tool Weblogo [45]), ‘Literature’ indicates the corresponding motif logos published in literature, ‘
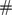
’ indicates the number of binding sites found by either FMotif or CisFinder, and ‘Rank’ after ‘
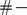
’ is the ranking number of a reported motif found by either FMotif or CisFinder.

**Figure 2:**
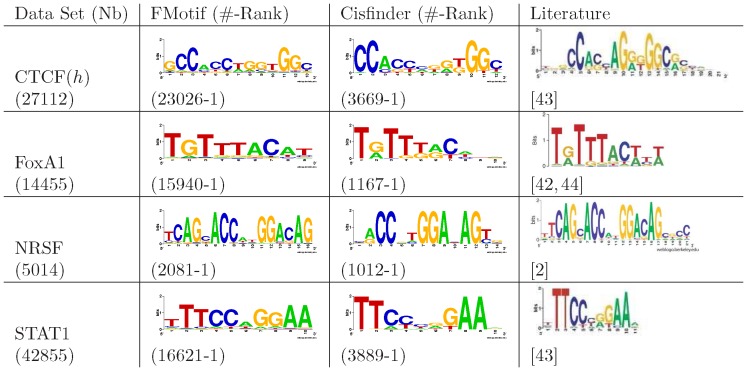
Motifs in 4 human TF ChIP-seq data sets. FMotif was tested with four widely used human ChIP-seq data sets for four DNA-binding TFs including CTCF (CCCTC-binding factor [41], named CTCF(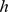)), FoxA1 (hepatocyte nuclear factor 3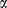 [42]), NRSF (neuron-restrictive silencer factor [2]), and STAT1 (signal transducer and activator of transcription protein [1]). Results from CisFinder and published motifs in literature are shown for comparison. Column definitions are the same as those in Figure 1.

In order to facilitate a fast motif search, avoid the potential influence of false positive peaks, and reduce false positive motifs in background sequences [37], we ran FMotif on the first 3000 ChIP-enriched genomic sequences and then scanned for potential binding locations in the remaining genomic sequences with the degenerative value 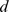. Since binding sites could exist on either DNA strand and CisFinder searched both, we counted the instances of a predicted motif and those of its reverse complement motif. We then compared FMotif with CisFinder and published motifs [2], [40], [42]–[44] in literature. The experimental results are shown in Figures 1 and 2, where ‘Nb’ indicates the number of peak-enriched regions predicted by the peak-calling program MACS with an FDR threshold of 0.2 or a 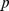-value threshold of 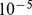, ‘FMotif’ and ‘CisFinder’ indicate the closest matching motif logos found by these programs (all motif logos were generated using the web-based tool Weblogo [45]), ‘Literature’ indicates the corresponding motif logos published in literature, ‘
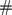
’ indicates the number of binding sites found by either FMotif or CisFinder, and ‘Rank’ after ‘
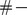
’ is the ranking number of a reported motif found by either FMotif or CisFinder.

We compared predicted and published motifs using a motif-level accuracy measure called the *performance coefficient* 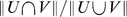, where 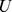 is a predicted motif consensus and 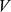 is the motif consensus published in literature [19]. As shown in Figures 1 and 2, the motif logos found by FMotif were more accurate compared with published logos from literature than those found by CisFinder. Furthermore, FMotif identified more TFBS locations than CisFinder. As for the 12 mouse TFs DNA-binding logos in [40], Chen et al. used the motif discovery algorithm WEEDER and subsequently extended the motifs using an expectation-maximization method. This second step was necessary because the supplied version of the WEEDER algorithm limited the motif search to a maximum of 12 bps. As discussed in the previous sections, WEEDER operated with low efficiency for long motifs and was difficult to tune for the parameter 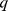.

To estimate the robustness of our sampling strategy, we ran FMotif on the first 500, 1000, 1500, …, and 5000 sequences and the full-size ChIP-enriched genomic sequences of TFs n-Myc, Oct4, and NRSF. For all subsets and the full-size data sets, each of the corresponding motifs in Figures 1 and 2 was ranked within the top 25 motifs predicted by FMotif. The ranking number of reported motifs increased with subset size and tended to be stable when the size was greater than 1000. All potential binding sites of reported motifs were obtained from subsets and discovered during the scanning step. Thus, it was not necessary to run a motif-finding algorithm on the whole ChIP-seq data sets, especially when data sets were very large. Additionally, we tested FMotif on 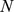 randomly selected sequences (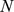 = 500, 1000, 1500, …, and 3000). These experiments were repeated 10 times. We then compared these results to those of the first 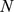 sequences and those of the last 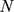 sequences for TFs n-Myc, Oct4, and NRSF. In general, motif consensuses predicted from the first 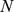 sequences were the most similar to published motifs and ranked highest in the final output. Those predicted from randomly selected 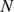 sequences tended to be ranked second, while those predicted from the last 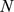 sequences were usually ranked the lowest. Furthermore, for the same reported motif of a TF, the number of binding sites found in the first 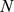 sequences was significantly greater than that found in randomly selected 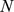 sequences. The number of predicted binding sites found in the last 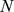 sequences was the lowest. In some cases there was no corresponding motif in randomly selected 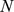 sequences or in the last 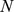 sequences when employing the same parameter settings. This situation occurred more often when using the last 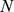 sequences. Therefore, we decided that the first 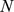 sequences with the lowest 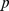-value or FDR (i.e., the most ChIP-enriched sequences) were the best choice for drawing statistical conclusions about a corresponding motif. This is because, as discussed in the Introduction section, the first 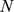 sequences were the least affected by noise. We selected the first 3000 peak regions to be sure that the selected subsets were large enough to account for the specificity of TF-DNA binding and to exclude false positive motifs. The same results may be obtained by running the algorithm on the first 1000–2000 sequences and then scanning potential locations in the remaining sequences.

### FMotif Sensitivity

**Figure 3:**
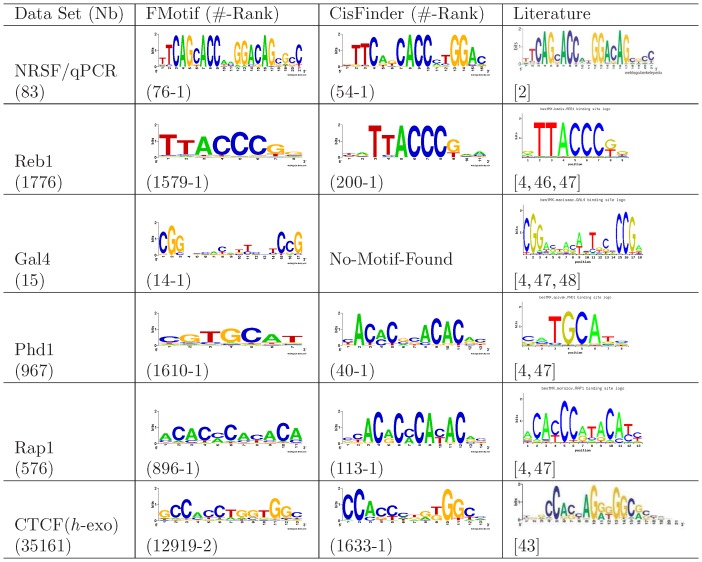
FMotif sensitivity. FMotif sensitivity was measured using an NRSF-positive TFBS set (NRSF/qPCR), which was composed of 83 binding sites verified by qPCR [2], four yeast DNA-binding TFs (Reb1, Gal4, Phd1, and Rap1), and one human TF (CTCF) ChIP-exo data sets. Results from CisFinder and published motifs in literature are shown for comparison. Column definitions are the same as those in Figure 1.

To test the sensitivity of FMotif, we ran it on an NRSF-positive TFBS set (NRSF/qPCR), which was composed of 83 binding sites verified by qPCR [2]. We then ran FMotif on four yeast DNA-binding TFs (Reb1, Gal4, Phd1, and Rap1) and one human TF (CTCF) ChIP-exo data sets. Raw sequence of the five ChIP-exo data sets are available from the NCBI Sequence Read Archive with accession number SRA0044886 [4]. Since it is thought that 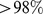 of ChIP-exo peak regions contain one recognizable DNA-binding motif within tens of bps away from peak summits, these can be viewed as positive TFBS sets. We used the five ChIP-exo peaks reported in Data S1 from Rhee and Pugh [4]. Similarly, we mapped the summits of ChIP-exo peaks and extracted 50 bps of genomic sequence centered around each peak in yeast genome sacCer3 and human genome hg19, respectively. This allowed us to avoid peak regions overlapping with each other due to some of the summits of ChIP-exo peaks being very close together. Results from CisFinder and published motifs [2], [4], [43], [46]–[48] in literature are shown for comparison (see Figure 3).

As shown in Figure 3, FMotif was capable of finding more matching motifs and true TF-binding locations when compared to CisFinder. For example, 76 true binding sites of NRSF/qPCR were predicted exactly by FMotif. On the same data set (NRSF/qPCR), MICSA [38] using MEME reported only 55 sites. This highlights the fact that FMotif is capable of identifying TF-binding locations with high sensitivity. It is well-established that specificity is an important consideration for this type of method. However, the ChIP-exo technique is a high-throughput approach, and the resolution of binding regions identified by ChIP-exo may still be tens of base pairs from where the true binding sites of between 8 and 25 base pairs are located. In addition, some of those binding regions are false positives, and it is difficult to say which ones are truly false positives without carefully designed wet-lab experiments. However, we show the specificity (i.e., 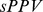) of FMotif for artificial samples in Tables 6 and 7. From this information we conclude that FMotif has both a higher sensitivity and a higher specificity than CisFinder.

## Discussion

In this study, we have proposed a new and fast heuristic enumeration method, FMotif, for extracting motifs from sequences. We have used this method to identify motifs and their binding locations in widely-used large-scale ChIP-seq and ChIP-exo data sets by combining FMotif with a peak-enriched sampling strategy. Our empirical studies have shown that this algorithm is fast and exact when searching for motifs in (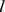, 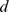) samples and has achieved good performance when identifying motifs in ChIP-enriched regions. In addition, the ChIP-enriched sampling strategy worked well on large-scale ChIP-seq and ChIP-exo data sets. It not only allowed us to exclude both noise occurring in lower ChIP-enriched peak regions and false positive motifs contained in background sequences, but also let us take advantage of well-developed motif-finding tools with low-level scalability. However, it should be pointed out that, in general, no method can outperform others under all conditions. FMotif performed faster than SPELLER, WEEDER, and MITRA but used more memory to store mismatched information in suffix trees, and FMotif was much more accurate but much slower than CisFinder. FMotif does, however, provide a good trade-off between time, space, and accuracy.

Motif discovery has been a popular area of study for more than two decades. Many successful motif-finding programs have been developed. The programs are ideal for finding motifs in tens or hundreds of promoters of co-regulated or homologous genes and for extracting motifs in genome-wide ChIP-enriched regions contained in large-scale ChIP-chip, ChIP-seq, and ChIP-exo data sets. Still, the problem is far from solved due to diversity in gene expression/regulation and the low specificity of binding sites. With the advance of high-throughput and high-resolution sequencing techniques like ChIP-exo, researchers have an increasing number of tools for studying gene regulation on a genomic scale. This will make the motif-finding problem easier to solve. Using advanced techniques such as ChIP-exo, it is possible to acquire new knowledge of regulatory binding sites. This will not only be beneficial for understanding the mechanisms of gene regulation, but also for creating a proper computational model that will replace (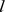, 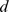) models and PWM matrix profiles for motif representation.

## Materials and Methods

### The (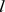, 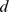) Motif Search and Suffix Tree

A transcription factor binds to specific DNA sequences and is involved in controlling the transcription of genetic information from DNA to mRNA. The actual DNA regions bound by a TF usually range in size from 8–10 to 16–20 bps and display a short motif, but differ by a few nucleotides from one another. The computational problem is to determine such a motif by analyzing a set of sequences that contain instances of the motif.

In current literature, there are two main approaches to motif representation. The first involves using a motif profile characterized by a PWM 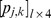. The PWM records the probability of an observed nucleotide 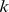 (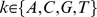) at position 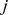 (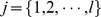) for all aligned sites, where 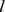 is the length of the motif. Numerous programs have been developed to maximize the score of a PWM by measuring, for example, the information content of a PWM:
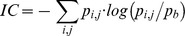
where 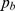 is the background frequency for the nucleotide, which measures motif conservation [7]. Using the second approach, one can characterize a motif as an 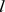-length consensus string and describe it using the most frequent nucleotide in each position of all aligned sites under the assumption that each sequence contains zero or one motif instance with up to 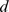 or exactly 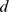 mutations within the motif. Finding (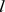, 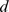) motifs with exactly 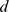 mutations is more challenging than finding (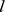, 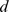) motifs with up to 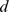 mutations, and algorithms designed for the former can usually be directly used to find the latter. In addition, profile-based optimization methods, e.g., CONSENSUS and MEME, have failed to find (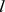, 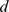) motifs such as (15, 4), where a 15-bp motif is planted into 20 sequences, each 600 bps in length with exactly 4 mismatches [19]. Thus, in this study we focus on designing a fast and exact algorithm to find (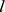, 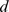) motifs with exactly 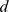 mutations on (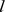, 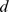) samples.

**Figure 4:**
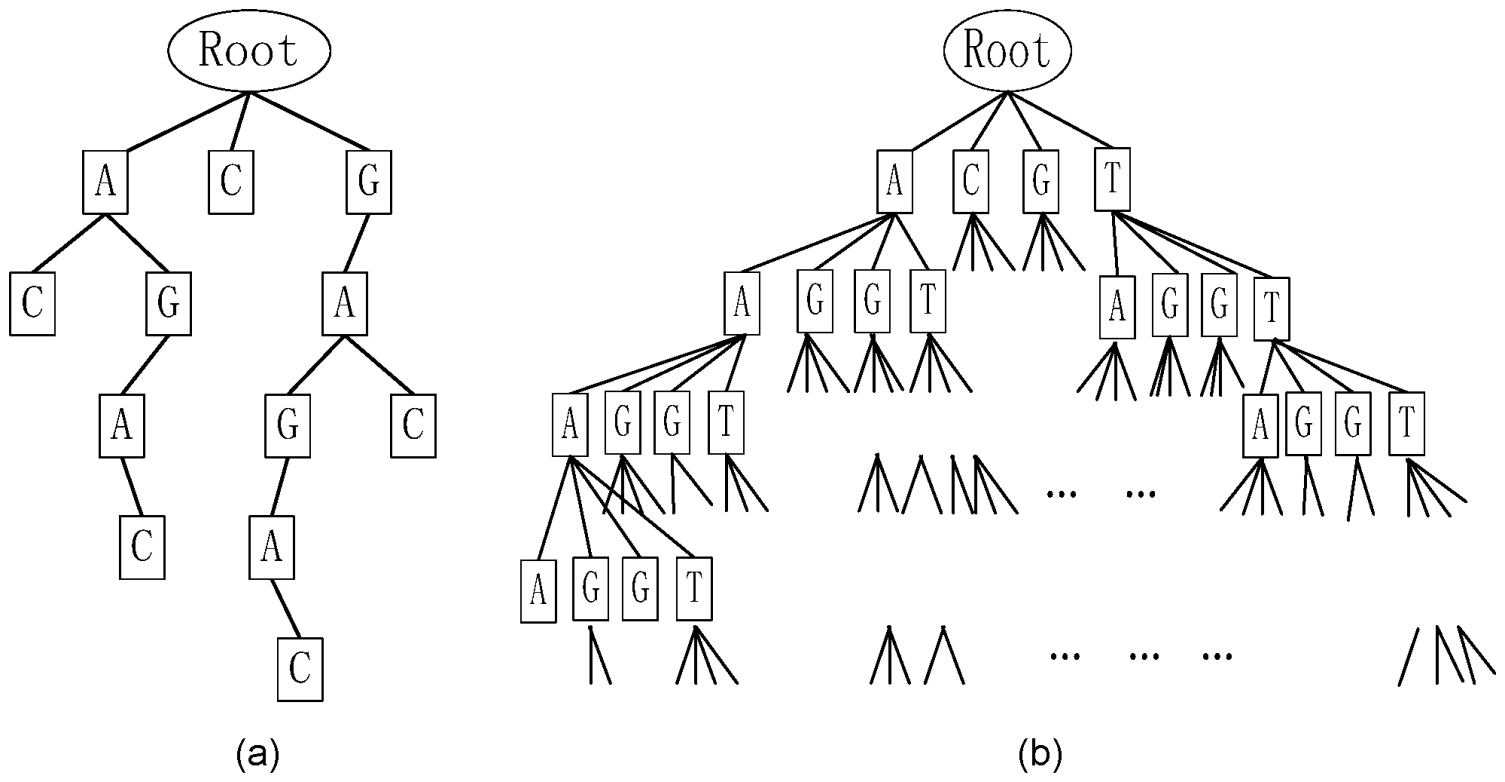
An example of a suffix tree and a tree representation of pattern space. (a) The suffix tree of the sequence GAGAC. (b) A tree representation of pattern space in the search for an (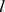, 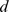) motif.

When searching for the exact motifs contained in an (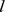, 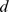) sample, it is customary to perform an exhaustive search for all potential 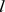-mers and verify their occurrence in the entire sample set. When using SPELLER and WEEDER to perform fast 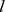-mer substring searching in a sequence set, a suffix tree structure is used to index sequences. A suffix tree presents the suffixes of a given string or a given set of strings in a way that allows for a very fast implementation of string operations. An example of a classic suffix tree for the string GAGAC is shown in Figure 4a. When the suffix tree of a string with length 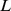 is constructed, searching for a substring of length 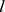 (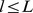) in the string only requires time proportional to 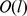 instead of 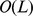.

Nevertheless, it is still time consuming to perform an exhaustive motif search in a suffix tree of sequences because the search space (shown as a four-branch tree in Figure 4b) can be up to 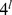 in size. With the increase in degenerative value 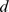, the valid instances of a motif in the suffix tree will increase dramatically. Therefore, SPELLER can handle only short (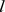, 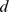) motifs with 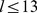 and 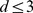. In order to increase the speed of SPELLER, WEEDER introduces an error ratio 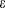 (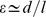) to narrow the search space such that for all 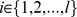, the number of mismatches between the first 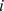 nucleotides of a candidate 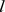-mer motif and the first 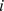 nucleotides of a valid instance of the motif is at most 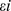. The strategy can quickly discard 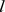-mers in the search space that do not satisfy this restriction. However, not all motif occurrences satisfy this restriction, and therefore the real motif may be missed by the algorithm. WEEDER lowers the occurrence frequency 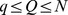 to make sure that the true motif will not be missed. Still, WEEDER is an almost exact algorithm. What’s more, with the decrease of 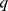, WEEDER’s run time will increase dramatically [14]. Therefore, a fast and exact (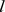, 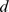) motif search method is needed.

### Mismatched Suffix Trees and FMotif

SPELLER and WEEDER use a depth-first search to scan the entire pattern space. If an 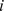-mer along the pattern tree has enough instances in the suffix tree of sequences, the 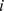-mer can grow up to 4 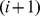-mers in the next layer of the pattern tree (see Figure 4b). Otherwise, the end node of an 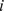-mer in the pattern tree will not be allowed to grow and the sibling nodes of the 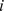-mer will be checked. If any of the sibling nodes can grow to the 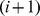-th layer in the pattern tree, the search process will go down to the 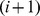-th layer of the pattern tree in a depth-first manner. Otherwise, it will backtrack to the uncle nodes (the siblings of parent nodes) of the 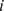-mer in the 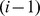-th layer of the pattern tree and so forth. The algorithms will end at the longest 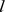-mer or 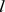-mers in the pattern tree. The difference between SPELLER and WEEDER is that WEEDER reduces the number of possible instances of a motif by restricting its mutation locations such that the valid paths on the pattern tree are sharply reduced.

We discovered that for finding motifs with length 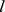, both SPELLER and WEEDER must check each 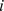-mer (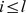) in the pattern space with depth-first order and count the variants of the 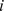-mer in the suffix tree of sequences from the root to layer 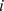. The suffix tree is scanned one time for each 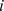-mer pattern. Thus, the algorithms scan the suffix tree an increasing number of times with the increase of 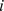. Actually, the mismatch information in layer 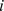 of a suffix tree can be used to search (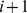)-mers in the pattern space. In this work we constructed a new suffix tree structure with mismatch information, called a mismatched suffix tree, for each sequence. Using these trees, we took advantage of the mismatch information in the 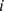-th layer of the trees to verify (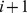)-mers in the pattern space and then updated the mismatch information in the 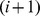-th layer. In this way we were able to implement a depth-first search on the pattern space and mismatched suffix trees simultaneously, which avoided a large number of repeated scans on the suffix trees of sequences.

**Figure 5:**
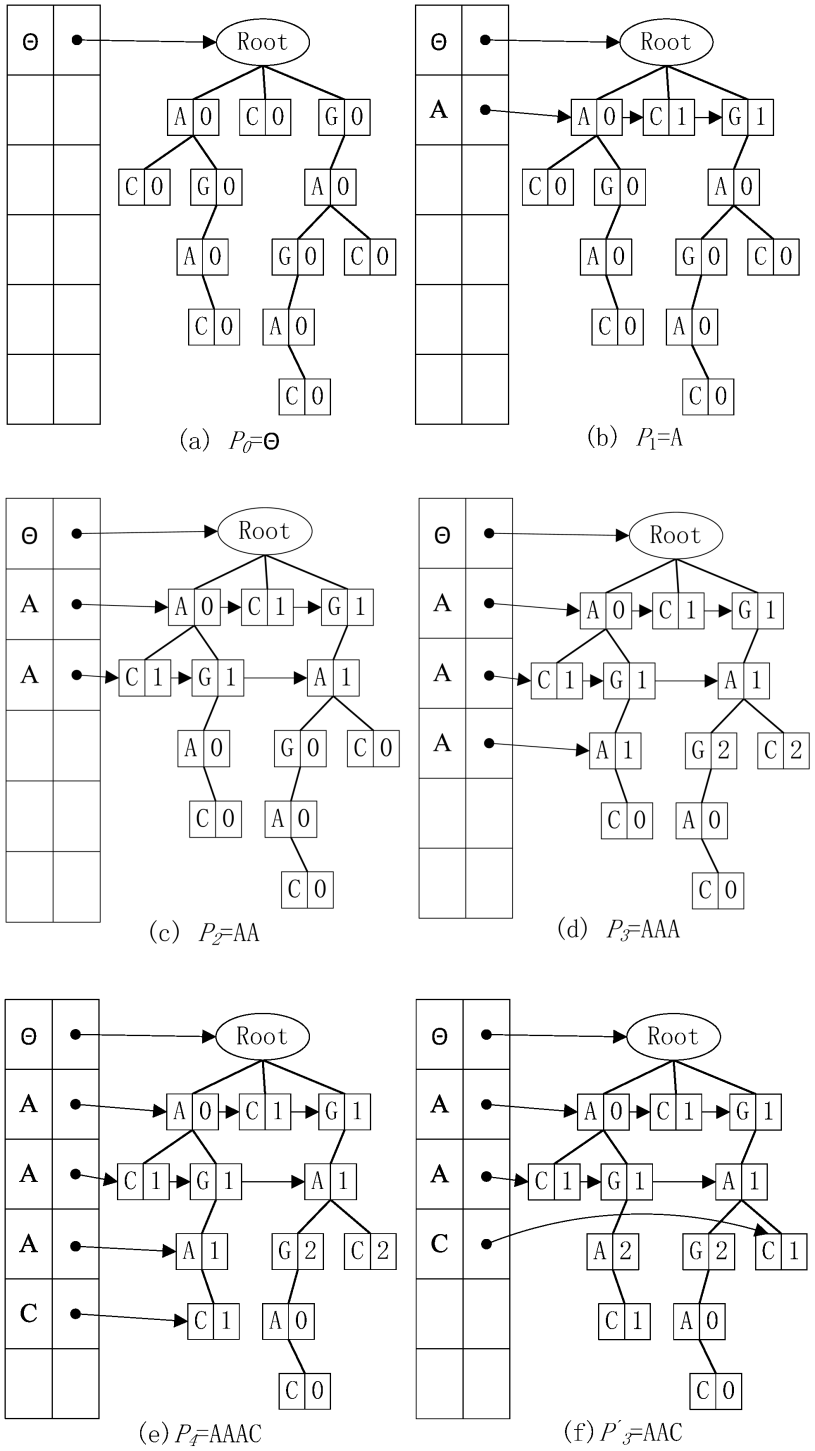
An example of a (4,1) motif search using FMotif. Figures (a)–(f) illustrate the search process of (4, 1) motifs on the mismatched suffix tree of the sequence GAGAC.


For instance, when searching occurrences of (4, 1) motifs in the sequence GAGAC, we started from the root of the pattern tree represented as 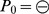 in Figure 5a and initialized the mismatched suffix tree for the sequence GAGAC. We then checked the occurrences of pattern 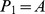 with up to 1 mismatch in the mismatched suffix tree and found that all nodes in the first layer have 0 or 1 mismatch(es) with 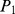. Next, we updated the mismatch value along the valid nodes and linked all of these nodes by points (see Figure 5b). We subsequently performed a depth-first search again and arrived at the pattern 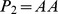. We updated mismatch information for all child nodes of the nodes in the link set in the first layer by using the mismatch information of those nodes in the link set and found all nodes in the second layer had 1 mismatch with the pattern 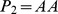. We updated the mismatch value along the valid nodes in the second layer and linked all of these nodes by points to form a new link set (see Figure 5c). Then, we moved to the pattern 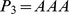 in a depth-first manner and updated mismatch information of all child nodes of the nodes in the newly generated link set by using the mismatch information of those nodes in the new link set. We found that only the child node A, representing the 3-mer AGA from the root to node A in the third layer of the suffix tree, had 1 mismatch with the pattern 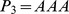 (see Figure 5d). Other nodes with 2 mismatches did not need to be updated and checked for the longer pattern 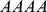. We found that the child node of the node A in the third layer did not satisfy the 1-mismatch restriction with the pattern 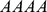, so we looked at the pattern 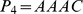 and found a (4, 1) occurrence of 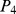 (see Figure 5e). We then went to the patterns 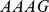 and 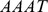 and found no occurrence of these patterns in the sequence GAGAC. We backtracked to the pattern 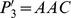 and updated the mismatch information in the third layer by using the mismatch information of their parent nodes in link set of the second layer. There we found that only node C in the third layer satisfies the restriction (see Figure 5f), but that node C has no child. We then backtracked to pattern 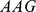 and continued the process as before until we found all occurrences for each (4, 1) motif. The details of the pattern search and mismatched suffix tree construction are shown in the subroutine 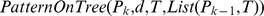, where 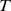 is the mismatched suffix tree for a sequence, 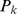 is the node currently being processed (representing a 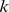-mer pattern) in the 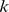-th layer of the pattern tree, 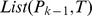 is the link set representing all valid occurrences of the 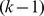-mer pattern represented by the node 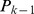 in the 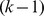-th layer of the pattern tree, and 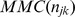 is mismatch value of the pattern represented by 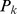 compared with the substring represented by the node 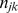 in the tree 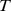.

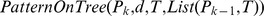


Initialize 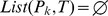;

**for** 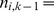
head node to tail node of 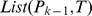 **do**

**for** each 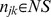 **do** (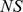 is the child node set of the node 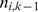)

**if** 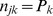, **then**


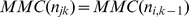


**else**


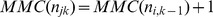
;

**if** 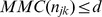 **then**

   add 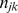 to 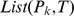;

**return** 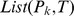.


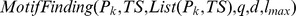


Initialize 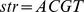;

**if** 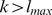 or 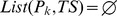 then

**return**;

**for** j = 1 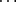 4 **do**


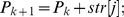



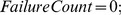


**for** each tree 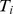 in the tree set 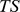 **do**


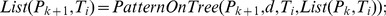


**if** 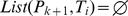 **then**


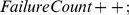


**if** 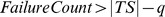 **then**

**break**;

**if** 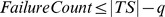 **then**


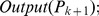



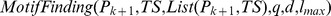


For each set of sequences, we counted the number of occurrences of a potential pattern in all sequences instead of just one sequence shown in subroutine 
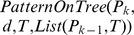
. If the number of occurrences was larger than the threshold of occurrence frequency 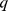, it was reported as a potential pattern. The subroutine for counting occurrences of a 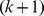-mer pattern, represented by the node 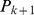 in the 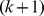-th layer of the pattern tree, is shown in 
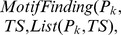
 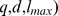, where 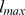 is the maximum length allowed for a motif, 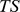 is the set of mismatched suffix trees for all sequences, 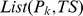 = 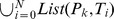, and 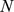 is the number of total sequences.

The entire process of finding motifs with at least 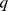 occurrences in a set of sequences is shown below. Additionally, since there may be many motifs that satisfy the quorum restriction 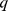, we sorted all potential motifs according to their statistical significance using the method in [14], [15]. We reported the top 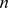 significant motifs and their occurrences as output, where (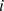, 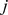) indicates an instance of a motif starting at the 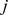-th position of the 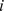-th sequence 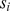.

### The FMotif Algorithm

1. Initialize a mismatched suffix tree 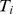 like the one shown in Figure 5a, 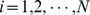;
2. Initialize 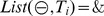
tempnode, where 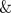
tempnode is the pointer of a temporary node, 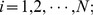

3. Input 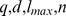;

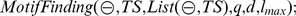

5. Rank the found motifs according to their significance scores;
6. Output the top 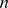 motifs, their instances, and the positions (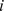, 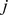) of these instances.

According to our empirical study, FMotif is capable of increasing the speed of the algorithms SPELLER and WEEDER without loss of accuracy. In addition, we used the WEEDER strategy to further decrease the search space by allowing mismatches occurring at most 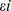 times with an increase in 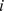. This strategy decreased FMotif’s run time but caused problems during the tuning of the parameter 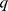 and resulted in a loss of accuracy.
